# Identification of key microRNAs in the carotid arteries of ApoE^−/−^ mice exposed to disturbed flow

**DOI:** 10.1186/s41065-019-0112-x

**Published:** 2019-11-05

**Authors:** Xinzhou Wang, Shuibo Gao, Liping Dai, Zhentao Wang, Hong Wu

**Affiliations:** 1 Laboratory of Cell Imaging, Henan University of Chinese Medicine, 6 Dongfeng Rd, Zhengzhou, 450002 Henan China; 2 School of Pharmacy, Henan University of Chinese Medicine, Zhengzhou, 450046 China; 3 Institute of Cardiovascular Disease, Henan University of Chinese Medicine, Zhengzhou, 450002 China

**Keywords:** Atherosclerosis, Oscillatory blood flow, microRNA, Microarray analysis, Bioinformatics

## Abstract

**Background:**

Atherosclerosis (AS) is one of the main causes of cardiovascular disease. AS plaques often occur in blood vessels with oscillatory blood flow and their formation can be regulated by microRNAs (miRNAs). The aim of this study is to identify the key miRNAs and molecular pathways involved in this pathological process.

**Methods:**

In this study, gene chip data obtained from the GEO database was analyzed using the LIMMA package to find differentially expressed miRNAs (DE miRNAs) in the carotid arteries of ApoE^−/−^ mice exposed to different blood flow rates. Predicted targets of the DE miRNAs were identified using the TargetScan, miRDB, and DIANA databases respectively, and the potential target genes (PTGs) were found by analyzing the common results of three databases. The DAVID database was used to enrich the PTGs based on gene ontology (GO) and pathway (Kyoto Encyclopedia of Genes and Genomes, KEGG), and the STRING database was used to uncover any protein-protein interactions (PPI) of the PTGs.

**Results:**

The networks of the DE miRNAs-PTGs, Pathway-PTGs-DE miRNAs, and PTGs PPI, were constructed using Cytoscape, and 11 up-regulated and 13 down-regulated DE miRNAs and 1479 PTGs were found. GO results showed that PTGs were significantly enriched in functions such as transcriptional regulation and DNA binding. KEGG results showed that PTGs were significantly enriched in inflammation-related mitogen-activated protein kinase (MAPK) and AS-related FOXO pathways. The PPI network revealed some key target genes in the PTGs.

**Conclusions:**

The analysis of key miRNAs and molecular pathways that regulate the formation of AS plaques induced by oscillatory blood flow will provide new ideas for AS treatment.

## Introduction

Cardiovascular diseases such as myocardial infarction have high morbidity and mortality rates, and atherosclerosis (AS) is the main pathological factor [1]. AS plaques are produced by the accumulation of lipids and cellulose in arteries, and are involved in pathological processes such as endothelial dysfunction, the accumulation of lipids and inflammatory cells, and remodeling of the extracellular matrix [2]. Hemodynamic signals play an important role in regulating vascular endothelial function. Disturbed-flow (D-flow) areas, such as bent or branched sites in arterial vessels, are more susceptible to developing AS plaques than stable-flow (S-flow) areas [3, 4]. As the vascular endothelium located at the bent or branched sites of the vessel is stimulated by oscillatory shear stress (OSS) in the D-flow environment, some adhesion factors and chemokines are up-regulated, leading to endothelial dysfunction. The S-flow maintains normal anticoagulant and anti-adhesion capabilities of the vascular endothelium through a stable laminar shear stress (LSS) signal [5, 6].

MicroRNAs (miRNAs) are a class of highly conserved, small, non-coding RNAs that can act as post-transcriptional regulators of genes [7]. In general, miRNAs first bind to the RNA-induced silencing complex (RISC), then preferentially bind to the 3′ untranslated region (3’UTR) of the target mRNA, and then the RISC can inhibit gene expression through mRNA degradation or inhibition of translation [8]. Studies have shown that multiple miRNAs can both accelerate, and prevent, the development of AS by interfering with target mRNA [9, 10]. miRNAs also play an important role in the development and progression of AS induced by blood flow shear stress [11, 12]. The roles of miRNAs in regulating AS under different shear stress are still unknown, and the interactions between miRNAs and their targets is unclear. In this study, we obtained the gene chip data of miRNAs expressed in the carotid arteries of ApoE^−/−^ mice under the intervention of differential blood flow shear stress from the GEO database, analyzed the differentially expressed miRNAs (DE miRNAs), and tried to find potential target genes (PTGs) of these DE miRNAs. By performing enrichment analyses on target gene function (GO) and pathway (Kyoto Encyclopedia of Genes and Genomes, KEGG), as well as PTGs protein-protein interaction (PPI), we have uncovered the molecular pathways and key miRNAs involved in the regulation of AS plaque formation induced by oscillatory blood flow, which will provide new targets for the treatment of AS.

## Materials and methods

### Gene chip data acquisition

Gene chip dataset GSE26555, miRNAs expressed in the carotid arteries of ApoE^−/−^ mice under different blood flow shear stress, was obtained from the GEO database (https://www.ncbi.nlm.nih.gov/geo/). Chip data samples were obtained from six 6–8 weeks old female ApoE^−/−^mice with their left carotid artery partially ligated. Mice were anesthetized by intraperitoneal injection of ketamine hydrochloride (80 mg/kg) and xylazine (5 mg/kg). The left external carotid, internal carotid, and occipital arteries were all ligated to ensure that blood flowed from the common carotid artery, out of the superior thyroid artery, resulting in a rapid decrease in the velocity of the blood flow of the common carotid artery, thereby reducing blood shear stress. After partial ligation of carotid artery, mice were fed a high-fat diet for 6 weeks. Left and right carotid arteries were collected and rinsed with 0.5–1 mL RNAlater solution. Total RNA was separated from both the partially ligated left and untreated right carotid arteries (control). The miRNA expression profile of the left carotid artery was compared with that extracted from the right carotid artery. 6 biological replicates were used per group with one replicate per array.

### Determination of DE miRNAs

The original data in the miRNA expression profile was converted into identifiable miRNA expression data according to the platform file. After normalization and log_2_ conversion, the data was processed using the LIMMA package (http://www.bioconductor.org/packages/release/bioc/html/limma.html). DE miRNAs were determined using these conditions: |log_2_ FC (fold change)| ≥1, adj *P* < 0.05.

### Construction of DE miRNAs-PTG network

TargetScan (http://www.targetscan.org/), miRDB (http://mirdb.org/), and DIANA (http://diana.imis.athena-innovation.gr/DianaTools/index.php?r=site/index) databases were used to predict the PTGs of DE miRNAs. PTGs were obtained using VENNY (https://bioinfogp.cnb.csic.es/tools/venny/) to extract the intersection of the predicted results from the three databases. Network maps of the DE miRNAs-PTGs were constructed using Cytoscape (available at https://cytoscape.org/), and the degree of each node in the maps was analyzed. To analyze the PTGs regulated by more than two DE miRNAs at the same time, a sub-network of DE miRNAs-PTGs was constructed using Cytoscape.

### PTGs function and pathway enrichment

PTG function and pathway enrichment analyses were completed using the DAVID database (https://david.ncifcrf.gov/). *P* < 0.05 was considered as statistically significant.

### Construction of PTGs interaction networks

The STRING database (https://string-db.org/) was used to obtain the PPI relationship between PTGs. Cytoscape was used to construct the PTGs-PPI network and to analyze the degree of each node.

### Statistics

TargetScan, miRDB and DIANA databases were used to predict the target genes of DE miRNAs under the set conditions respectively (cumulative weighted context^2+^ score < − 0.2, Target Score > 50, miTG score > 0.7). Pathway enrichment was set at *P* < 0.05 with significant difference. STRING database is used to obtain PPI relationship between PTGs under the condition (combined score > 0.4).

## Results

### Identification of DE miRNAs in carotid arteries of ApoE^−/−^ mice under different rates of blood flow shear stress

**Fig. 1:**
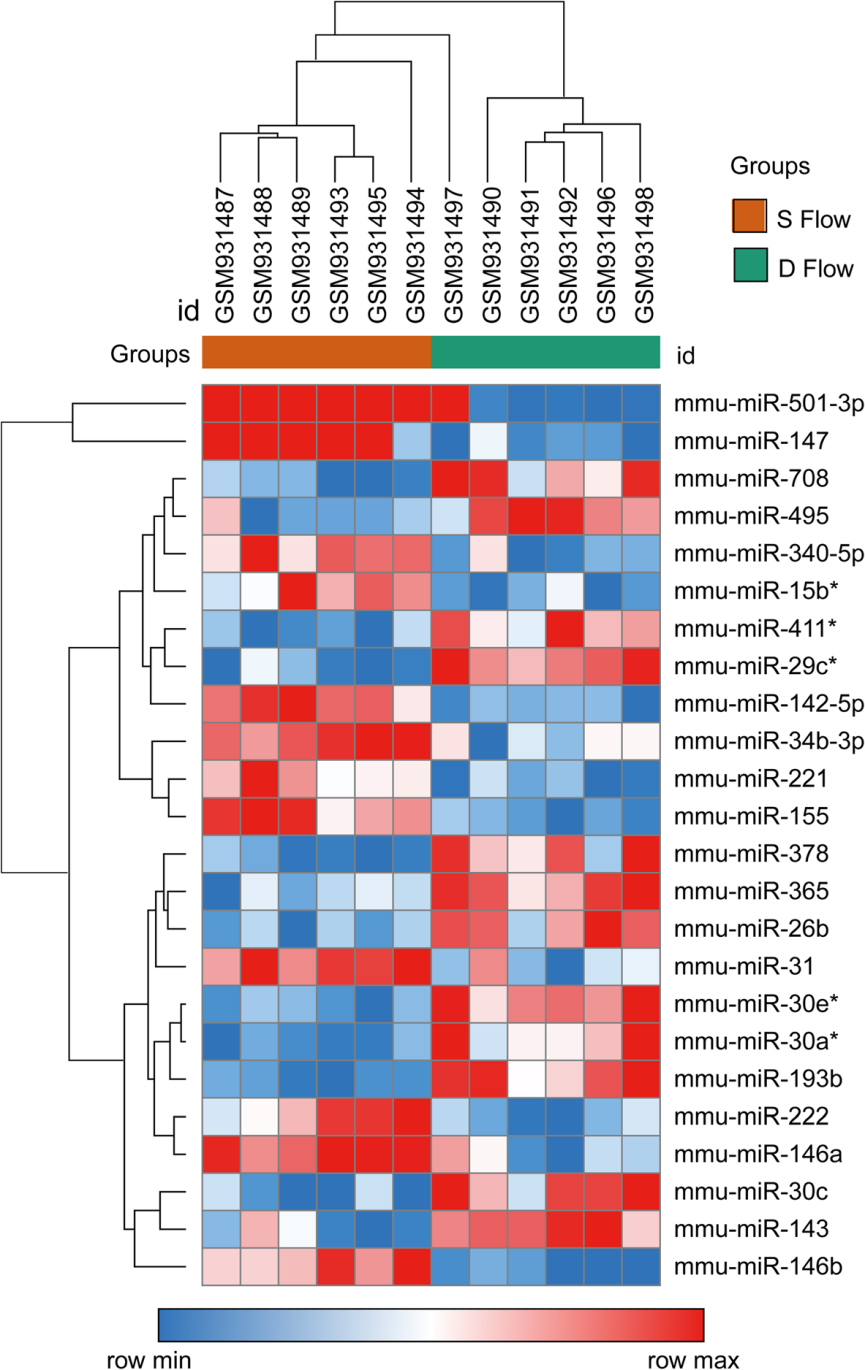
Heat map of DE miRNAs. Red represents up-regulation and blue represents down-regulation

Gene chip datasets were obtained from ApoE^−/−^ mice which had partially ligated left and untreated right carotid arteries. The blood flow in the untreated carotid artery was LSS, and the blood flow in the partially ligated carotid artery was OSS. To identify DE miRNAs from the normal and ligated carotid artery samples, the LIMMA package was used to analyze the expression profile. Twenty-four DE miRNAs were identified with the criteria of |log_2_ FC (fold change)| ≥1, and adj *P* < 0.05, 11 of which were up-regulated and 13 were down-regulated (Table 1). The heat map of DE miRNAs is shown in Fig. 1.

**Table 1:** Twenty four DE microRNAs induced by D-Flow

Probe ID	miRNAs	Log fold change	adj *P* value
mmu-miR-193b-4,395,597	mmu-miR-193b	1.477944	0.002299
mmu-miR-30e*-4,373,057	mmu-miR-30e*	1.072765	0.002972
mmu-miR-29c*-4,381,131	mmu-miR-29c*	1.414389	0.002972
mmu-miR-378-4,395,354	mmu-miR-378	2.007949	0.014718
mmu-miR-411*-4,395,349	mmu-miR-411*	1.040167	0.014718
mmu-miR-708-4,395,452	mmu-miR-708	1.148139	0.014718
mmu-miR-365-4,373,194	mmu-miR-365	1.840429	0.014718
mmu-miR-143-4,395,360	mmu-miR-143	2.066674	0.019814
mmu-miR-701-4,381,058	mmu-miR-701	1.235653	0.027888
mmu-miR-107-4,373,154	mmu-miR-107	1.878285	0.027888
mmu-miR-375-4,373,027	mmu-miR-375	3.853097	0.042681
mmu-miR-142-5p-4,395,359	mmu-miR-142-5p	−1.81988	0.002299
mmu-miR-146b-4,373,178	mmu-miR-146b	−2.98862	0.002299
mmu-miR-155-4,395,701	mmu-miR-155	−2.49596	0.002972
mmu-miR-147-4,395,373	mmu-miR-147	−8.1359	0.014533
mmu-miR-501-3p-4,381,069	mmu-miR-501-3p	−12.4471	0.014718
mmu-miR-146a-4,373,132	mmu-miR-146a	−1.6904	0.014718
mmu-miR-34b-3p-4,395,748	mmu-miR-34b-3p	−2.63985	0.014718
mmu-miR-221-4,373,077	mmu-miR-221	−1.82872	0.017999
mmu-miR-15b-4,373,122	mmu-miR-15b*	−2.04548	0.021745
mmu-miR-31-4,373,331	mmu-miR-31	−1.06863	0.021745
mmu-miR-222-4,395,387	mmu-miR-222	−1.52506	0.027568
mmu-miR-34c-4,373,036	mmu-miR-34c	−2.61381	0.027568
mmu-miR-146b*-4,395,583	mmu-miR-146b*	−8.907	0.027888

Note: miRNA*, miRNA-3p

### Construction of DE miRNAs-PTGs networks

**Fig. 2:**
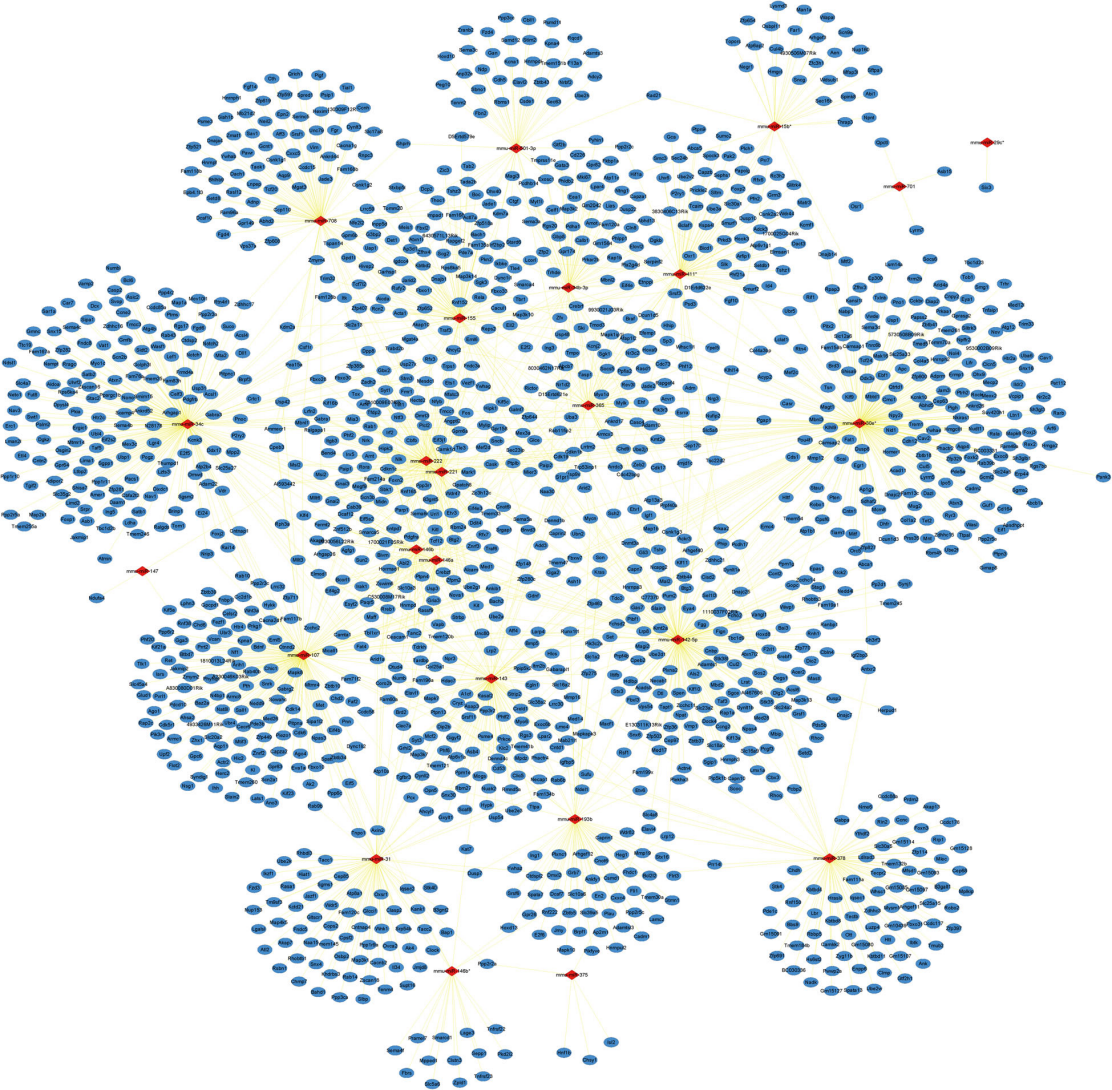
DE miRNAs-PTGs network. Diamond nodes represent DE miRNAs; circular nodes represent PTGs; straight lines represent the relationship between two nodes. DE miRNAs, differentially expressed miRNAs; PTGs, potential target genes

**Fig. 3:**
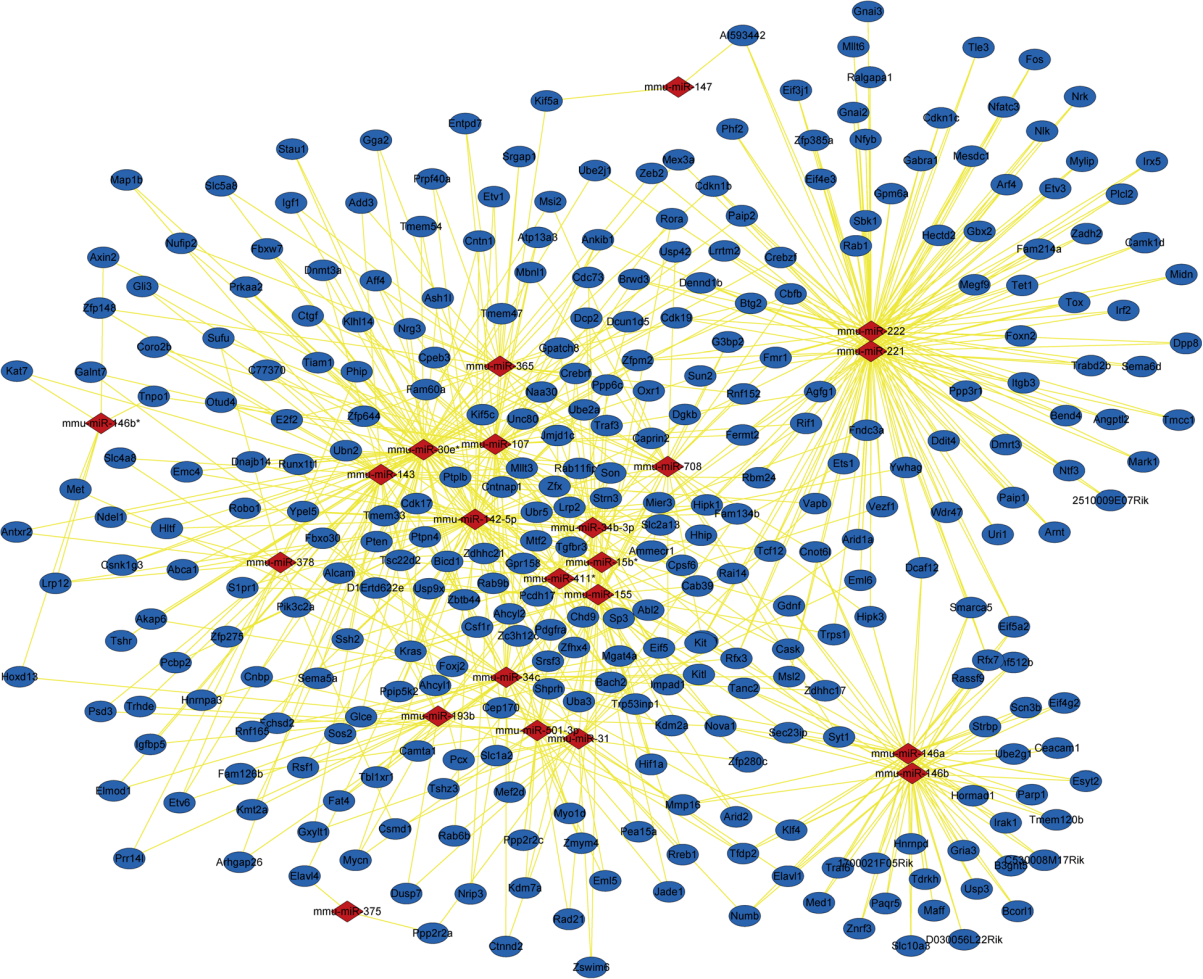
DE miRNAs-PTGs subnetwork. Diamond nodes represent DE miRNAs; circular nodes represent PTGs; straight lines represent the relationship between two nodes. Each PTG in the figure is regulated by at least two DE miRNAs. DE miRNAs: differentially expressed miRNAs; PTGs: potential target genes

miRNAs play a regulatory role in biological systems by interfering with mRNAs. To further analyze the target of DE miRNAs, the TargetScan, miRDB, and DIANA databases were used to identify the target genes of the twenty-four DE miRNAs. The results showed that there were 1479 PTGs and the network map of DE miRNAs-PTGs was constructed by Cytoscape (Fig. 2). The degree of each node in the network was calculated. Mmu-miR-30e*, mmu-miR-34c, mmu-miR-142-5p, mmu-miR-107, mmu-miR-143, mmu-miR-155, mmu-miR-221, mmu-miR-222, mmu-miR-378, mmu-miR-708 were identified as the 10 DE miRNAs with the highest degree (Table 2). A network between PTGs with degree≥2 and their corresponding DE miRNAs was constructed (Fig. 3). From the map, we found that the 5 pairs of DE miRNAs with the highest number of common target proteins were mmu-mir-221 & mmu-mir-222 (86), mmu-mir-146a & mmu-mir-146b (45), mmu-mir-30e* & mmu-mir-142-5p (15), mmu-mir-30e* & mmu-mir-143 (10), and mmu-mir-107 & mmu-mir-142- 5p (9).

**Table 2:** Ten DE miRNAs with the highest degree in DE miRNAs-PTGs network

Rank	DE miRNA-PTGs network		
	MiRNAs	Degree	Feature
1	mmu-miR-30e*	236	Up
2	mmu-miR-34c	190	Down
3	mmu-miR-142-5p	188	Down
4	mmu-miR-107	165	Up
5	mmu-miR-143	109	Up
6	mmu-miR-155	99	Down
7	mmu-miR-221	96	Down
8	mmu-miR-222	90	Down
9	mmu-miR-378	88	Up
10	mmu-miR-708	87	Up

Note: miRNA*, miRNA-3p

### PTGs function and pathway enrichment

**Fig. 4:**
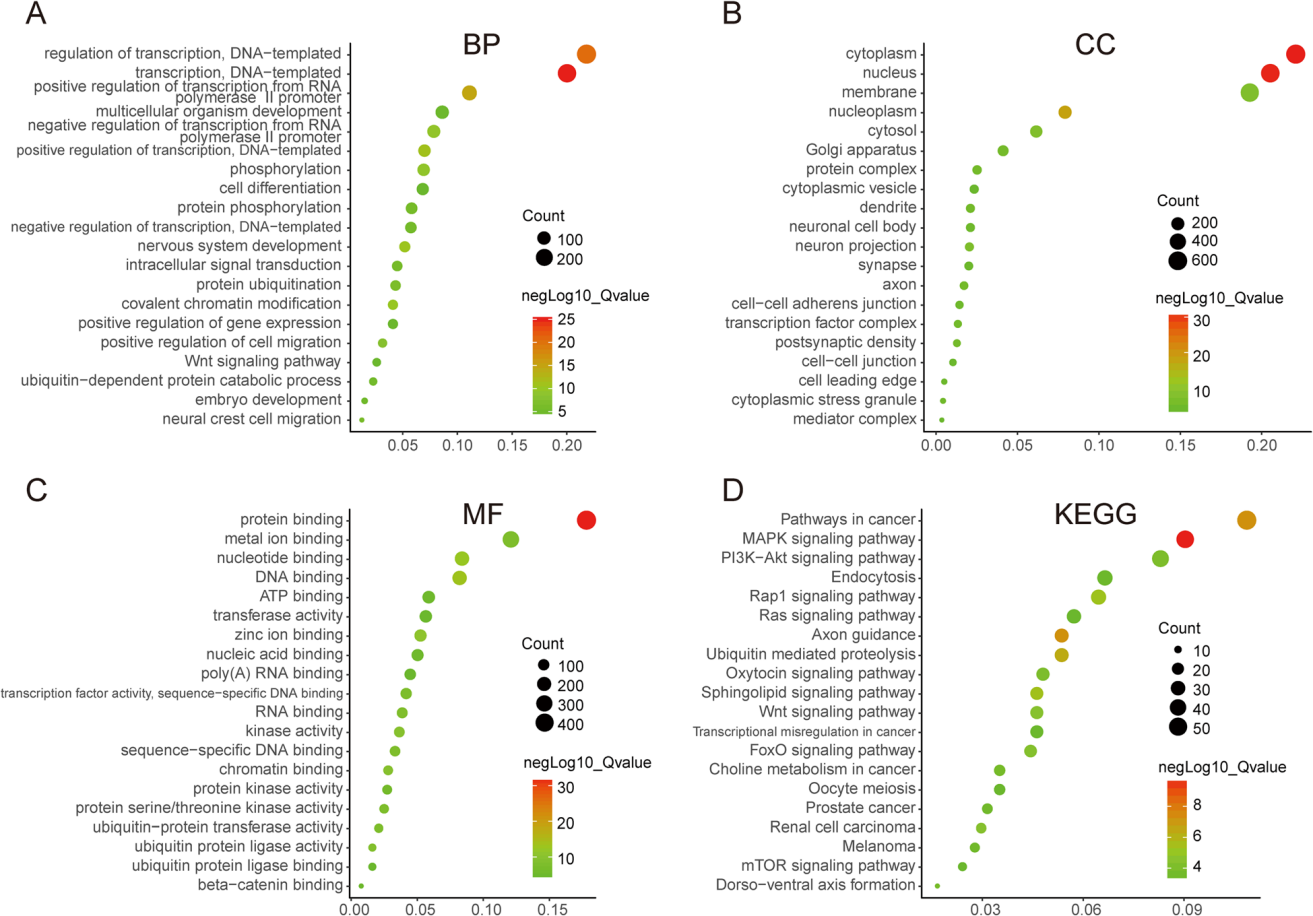
GO and KEGG enrichment analyses of PTGs. **a-c**: GO enrichment results; **d**: KEGG enrichment results. PTGs: potential target genes

**Fig. 5:**
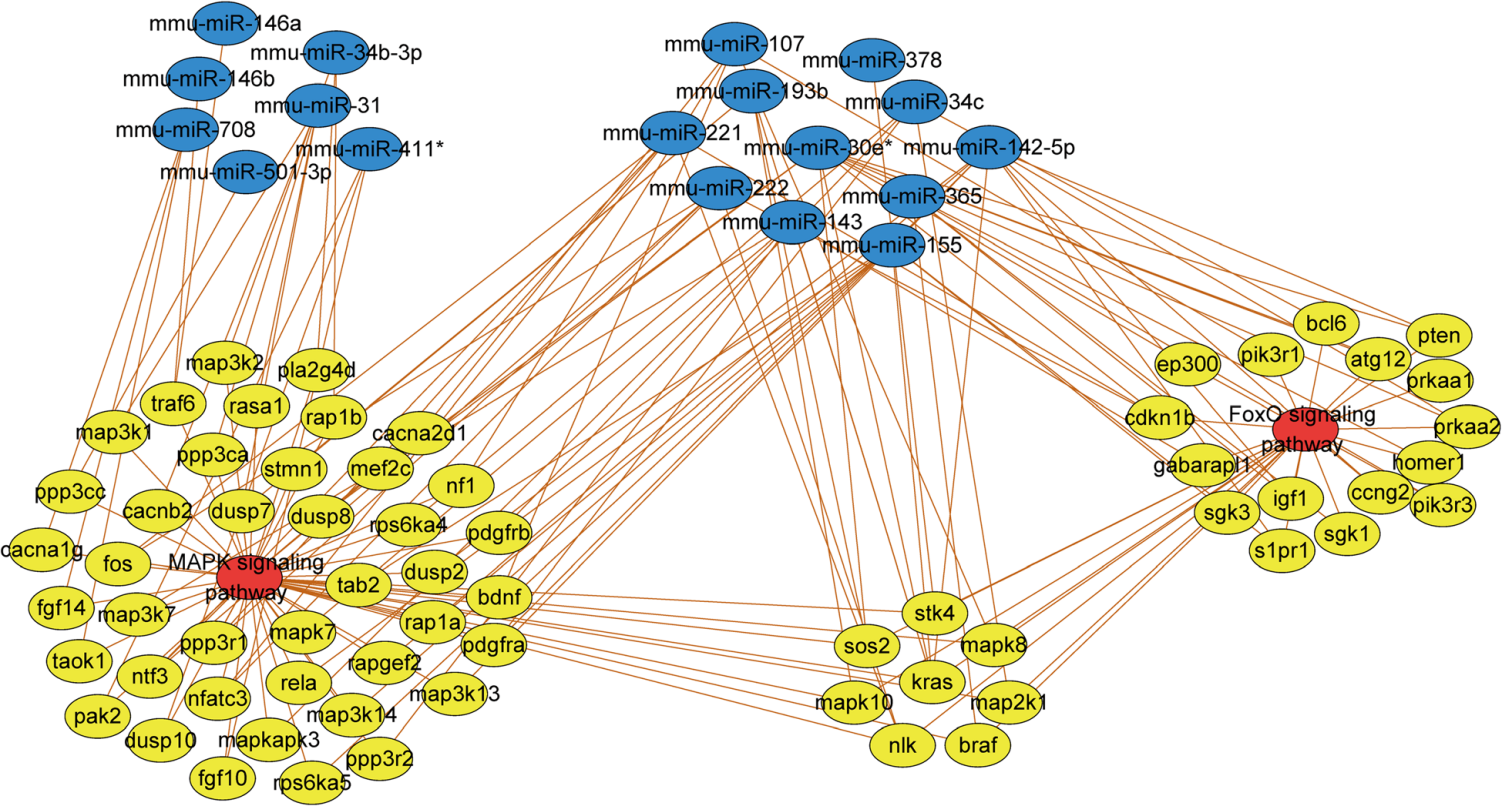
Network of MAPK and FOXO signaling pathways and related DE miRNAs-PTGs. Red nodes represent the signal path; yellow nodes represent genes; blue nodes represent miRNAs; and straight lines represent the relationship between two nodes. DE miRNAs: differentially expressed miRNAs; PTGs: potential target genes

Analysis of PTGs function and pathway enrichment is essential to understand the mechanism of target proteins. The GO enrichment results (Fig. 4A-4C) showed a significant enrichment of PTGs in gene transcriptional regulation and nucleic acid binding. The KEGG pathway enrichment results (Fig. 4D) showed PTGs were significantly enriched in the inflammation-associated mitogen-activated protein kinase (MAPK) signaling pathway, and the FOXO pathway, which is associated with AS formation. The network of MAPK and FOXO pathways and related PTGs and DE miRNAs, show that the regulation of DE miRNAs such as mmu-mir-107, mmu-mir-142-5p, mmu-mir-143 and mmu-mir-155 may be related to MAPK and FOXO pathways (Fig. 5).

### PTGs PPI network analysis

**Fig. 6:**
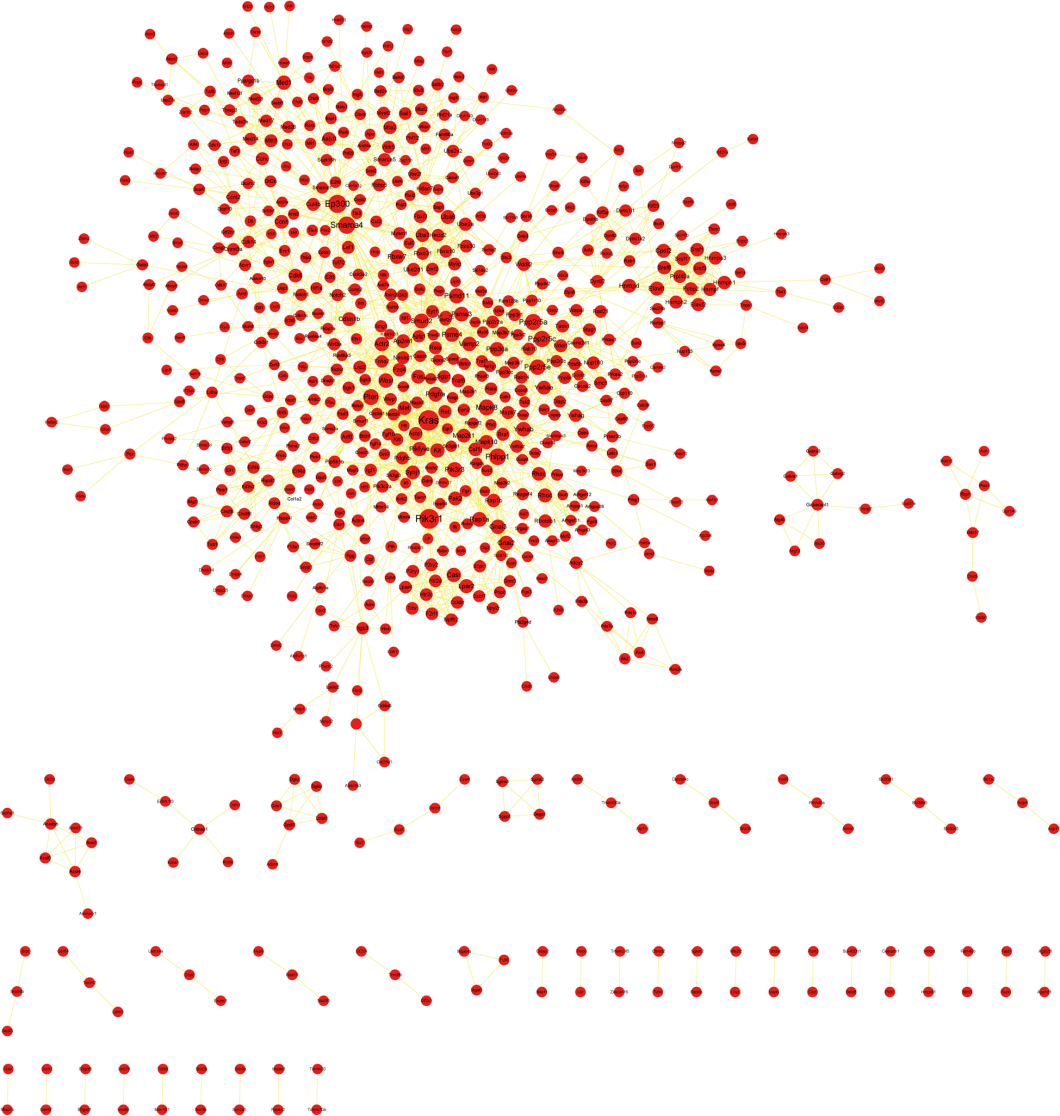
PTGs PPI network. Red nodes represent genes; straight lines represent the relationship between two nodes. Node size represents the degree; the bigger the node, the higher the degree

Proteins interact with each other to form a network which can influence biological functions. To examine key targets in this network, the STRING database was used to analyze the PPI of PTGs, the results were imported into Cytoscape to produce a PPI network map, and the degree of each node was calculated. The ten nodes with the highest degree were Kras, Pik3r1, Ep300, Smarca4, Phlpp1, Ppp2r5c, Ppp2r5a, Mapk8, Pten, and Ppp2r5e (Fig. 6).

## Discussion

In this study, we systematically analyzed the gene chip data of the ligated carotid artery of ApoE^−/−^ mice, and discovered the network of miRNAs and their targets involved in the formation of AS plaques induced by abnormal blood flow. Among them, 11 up-regulated and 13 down-regulated miRNAs with significant differences in expression levels were detected.

The theory that local hemodynamic changes could induce AS plaque formation has been proposed for decades, but the regulatory mechanisms involved are unclear. In recent years, more studies have been focused on the miRNA regulation of physiology and pathology. In AS development and progression, miRNAs play important roles including regulating the development of inflammation, immune cell adhesion, and lipid metabolism [13–16]. Although a direct link has been established between D-flow-induced AS formation and miRNAs regulation, a more comprehensive regulatory network has yet to be constructed.

miRNAs are typically transcribed by RNA polymerase II (RNA pol II) in the nucleus, first into pri-miRNAs which are then cleaved and processed in multiple steps to form double-stranded 20-25 nt miRNAs. The 20-25 nt miRNAs are further processed into two mature miRNAs, miRNA-5p and miRNA* (miRNA-3p). The seed sequence (2–8 nucleotides) of a mature miRNA specifically binds to the 3’UTR region of the target mRNA facilitating its cleavage and/or translational inhibition [17]. A single miRNA can regulate the expression of hundreds of genes and is biologically conserved [18]. The results of this study showed that there was a significant change in the expression of twenty-four mature miRNAs involved in the formation of AS plaques induced by oscillatory blood flow. Studies have shown that mir-29, mir-30, mir-34, mir-142, mir-143, mir-146, mir-147, mir-155, mir-221 and mir-222 are all associated with AS plaque formation [9, 11, 12]. Moreover, mir-34, mir-143/145 and mir-155’s direct regulation of the formation of AS plaques induced by D-flow has been confirmed [19, 20]. Mir-34 participates in endothelial cell inflammation induced by OSS. Blocking mir-34 expression can significantly reduce expression of adhesion factors VCAM-1 and ICAM-1. LSS promotes the expression of mir-143/145 in an AMPK alpha 2 and KLF2 dependent manner, and endothelial cell-derived mir-143/145 can be transported to medial smooth muscle cells through extracellular vesicles, thereby preventing AS formation by reducing their differentiation [21, 22]. In endothelial cells, LSS can induce the expression of mir-155, which is abundantly expressed in the intima of the thoracic aorta. Since the thoracic aorta is exposed to S-flow, this suggests that mir-155 is an anti-AS miRNA related to blood flow [23].

We further identified the PTGs of the DE miRNAs using the intersection of the predicted results from the three databases, built an interaction network, and found that 319 of the 1479 PTGs were regulated by two or more miRNAs, indicating that these DE miRNAs were functionally related. The 10 DE miRNAs in the network with the highest degree were mmu-mir-30e*, mmu-mir-34c, mmu-mir-142-5p, mmu-mir-107, mmu-mir-143, mmu-mir-155, mmu-mir-221, mmu-mir-222, mmu-mir-378, and mmu-mir-708. A High Degree reflects the key role of these miRNAs in this regulatory network, and most of these miRNAs have been shown to be associated with AS formation. The PPI network constructed from the PTGs shows the potential regulatory relationship between these gene targets. The ten highest PTGs of Degree were Kras, Pik3r1, Ep300, Smarca4, Phlpp1, Ppp2r5c, Ppp2r5a, Mapk8, Pten and Ppp2r5e, which were related to the proliferation of vascular smooth muscle cells and lipid metabolism in vivo [24–26]. These physiological disorders are closely related to AS [27, 28]. It is worth noting that the target genes of the DE miRNAs include KLF4, KLF9, KLF10, KLF11, and other KLF family members. Several members of the KLF family have been identified as transcription factors that regulate endothelial cell function-related genes [29–32]. GO results showed that PTGs were significantly enriched in functions such as transcriptional regulation and DNA binding, which is consistent with the above-mentioned results. Previous studies have used bioinformatics methods to analyze genes affected by D-flow in the carotid artery of mice [33]. Their modeling methods were similar to those used in this study and the DE mRNAs and some corresponding proteins were verified. This study focuses on miRNA regulation and predicts the PTGs and related pathways, as well as constructing a miR- target gene network. We will be able to validate these miRNAs in future studies.

AS was previously thought to be a lipid storage disease, but it actually involves a sustained inflammatory response. AS preferentially occurs at bent or branched sites in arteries, where the shear stress is low and oscillation is present [34, 35]. OSS can up-regulate the expression of endothelial pro-inflammatory factors and enhance the adhesion of circulating monocytes to the endothelium. In contrast, LSS inhibits the expression of pro-inflammatory factors and has a protective effect towards AS [36, 37]. The MAPK signaling pathway is involved in the regulation of AS inflammatory response and mice with MAPK signaling molecules knocked out showed significantly reduced AS lesions in experiment [38]. Normally, extracellular factors activate the MAPK pathway through a series of signal transductions which leads to the activation of the nuclear factor NF-κB and the upregulation of inflammatory factors [39, 40]. In this study, the KEGG pathway analysis on all PTGs revealed that the genes which were highly enriched in the MAPK pathway and the DE miRNAs involved in regulating these genes were mmu-mir-107, mmu-mir-142-5p, mmu-mir-143, mmu-mir-146a, mmu-mir-146b, mmu-mir-155, etc. These miRNAs could regulate the development of inflammation and AS through the MAPK pathway.

The FOXO family of proteins plays an important role in regulating the physiological functions of the biological system, among which, FOXO1 is highly expressed in vascular endothelial cells [41]. FOXO1 inhibits the transcription of endothelial nitric oxide synthase [42] and upregulates the expression of inducible nitric oxide synthase to cope with oxidative stress which leads to the production of peroxynitrite and endothelial dysfunction, and thereby promotes AS formation [43]. FOXO1 regulates the expression of the downstream transcription factor KLF2 in endothelial cells. KLF2 is a key factor in maintaining normal endothelial cell function and inhibits the formation of AS. The KEGG analysis in this study showed that PTGs were significantly enriched in the FOXO pathway, including mmu-mir-107, mmu-mir-142-5p, mmu-mir-143, mmu-mir-155, etc., and these miRNAs are also enriched in the MAPK pathway.

## Conclusion

In this study, we analyzed the gene chip data of miRNAs expression profiles in ApoE^−/−^ mice’s carotid arteries, and identified twenty-four DE miRNAs and their PTGs, and revealed that these PTGs were significantly enriched in transcription regulation, DNA binding, and inflammation-related, AS-related MAPK and FOXO pathways. The results provided new avenues for studying the molecular mechanism of AS plaque formation and designing targeted drugs.

## Data Availability

The database is available at https://www.ncbi.nlm.nih.gov/geo/. The GEO database is open access and all files described here are available through the website.

## Abbreviations

3’UTR: 3′ untranslated region
AS: Atherosclerosis
DE miRNAs: differentially expressed miRNAs
D-flow: Disturbed-flow
FC: Fold change
GO: Gene ontology
KEGG: Kyoto Encyclopedia of Genes and Genomes
LSS: Laminar shear stress
MAPK: Mitogen-activated protein kinase
miRNA: microRNA
OSS: Oscillatory shear stress
PPI: Protein-protein interactions
PTGs: Potential target genes
RISC: RNA-induced silencing complex
RNA pol II: RNA polymerase II
S-flow: Stable-flow
